# Repulsive guidance molecules b (RGMb): molecular mechanism, function and role in diseases

**DOI:** 10.1017/erm.2024.24

**Published:** 2024-10-08

**Authors:** Jie Zhang, Yijing Jiang, Zijian Zhang, Shilin Li, Haowen Fan, Jinhua Gu, Renfang Mao, Xiaohong Xu

**Affiliations:** 1 Department of Oncology, Affiliated Tumor Hospital of Nantong University, Nantong University, Nantong, Jiangsu, People's Republic of China; 2 Department of Pathophysiology, School of Medicine, Nantong University, Nantong, Jiangsu, People's Republic of China; 3 Nantong Institute of Genetics and Reproductive Medicine, Affiliated Maternity & Child Healthcare Hospital of Nantong University, Nantong, Jiangsu, China

**Keywords:** bone morphogenetic protein, cancer, diseases, programmed death-ligand 2, repulsive guidance molecules b

## Abstract

Repulsive guidance molecule b (RGMb), a glycosylphosphatidylinositol-anchored member of the RGM family, is initially identified as a co-receptor of bone morphogenetic protein (BMP) in the nervous system. The expression of RGMb is transcriptionally regulated by dorsal root ganglion 11 (DRG11), which is a transcription factor expressed in embryonic DRG and dorsal horn neurons and plays an important role in the development of sensory circuits. RGMb is involved in important physiological processes such as embryonic development, immune response, intercellular adhesion and tumorigenesis. Furthermore, RGMb is mainly involved in the regulation of RGMb–neogenin–Rho and BMP signalling pathways. The recent discovery of programmed death-ligand 2 (PD-L2)–RGMb binding reveals that the cell signalling network and functional regulation centred on RGMb are extremely complex. The latest report suggests that down-regulation of the PD-L2–RGMb pathway in the gut microbiota promotes an anti-tumour immune response, which defines a potentially effective immune strategy. However, the biological function of RGMb in a variety of human diseases has not been fully determined, and will remain an active research field. This article reviews the properties and functions of RGMb, focusing on its role under various physiological and pathological conditions.

## Introduction

Repulsive guidance molecules (RGMs) found in most vertebrate species are a family of cell membrane-associated proteins linked by glycosylphosphatidylinositol (GPI), including RGMa, RGMb, RGMc (Refs 1, 2, 3). Current studies have confirmed that the RGM family members in vertebrates include RGMa, RGMb and RGMc, while each genome of invertebrates encodes only one RGM protein (Ref. 4). The RGM family is a protein mainly present in the developing and adult central nervous system, named after the axon guidance molecule RGMa. As a member of the RGM famliy, RGMb is originally discovered in the study of factors regulated by dorsal root ganglion 11 (DRG11), and it is co-localized with DRG11 mRNA in DRG neurons and spinal cord (Ref. 5). Meanwhile, RGMb mRNA is also detected in developing neural tubes prior to the onset of DRG11 expression, and found in other regions of the nervous system that do not produce DRG11. With the discovery of the expression of RGMb in various tissues and organs, the regulatory effect of RGMb on important physiological and pathological physiological processes such as immune function, embryonic development, injury repair, intercellular adhesion, epithelial function maintenance and tumorigenesis have been paid more and more attention (Refs 6, 7, 8, 9, 10, 11, 12, 13). RGMb regulates a variety of physiological processes mainly through neogenin–Rho and bone morphogenetic protein (BMP) signalling pathways (Refs 14, 15, 16, 17). In addition, RGMb can interact directly or indirectly with other molecules such as programmed death-ligand 2 (PD-L2) to participate in a variety of important potential functions, which is one of the reasons why it has been paid attention by researchers (Ref. 18). Although the past 20 years have provided new insights into the role of RGMb in some diseases, especially tumours, there are still many physiological mechanisms involved in the development of diseases that need to be further clarified. This article will review the discovery, molecular structure, expression location, signalling pathway, physiological function and possible mechanism of RGMb in diseases.

## Sequence and structure

RGMb is a member of the RGM family and a single-stranded GPI-anchored membrane binding protein in mammals. GPI-anchored proteins are a kind of proteins that are anchored on the surface of eukaryotic cell membranes through their own carboxyl-terminal glycosyl-phosphatidylinositol structure but do not cross the phospholipid membrane bilayer (Refs 1, 19, 20). RGMb and other members RGMa and RGMc constitute the RGM family, and the homology between the three is more than 50% (Ref. 21). The common structural features of the RGM family include an N-terminal signal peptide, a partial von Willebrand factor D-type domain (vWF-type D), a hydrophobic domain with unknown function, and a C-terminal GPI anchoring domain. There is a highly conserved proteolytic cleavage site in the vWF-type D domain, which has not been found in other proteins. Studies suggest that vWF-type D cleaves the RGMs internal protein into two fragments, the N-terminus and the C-terminus containing the GPI-anchored sequence. The N-terminal domain is a conventional signal peptide, and the C-terminal domain is related to the characteristics of GPI-anchored membrane binding protein (Refs 17, 19, 22).

**Figure 1.**
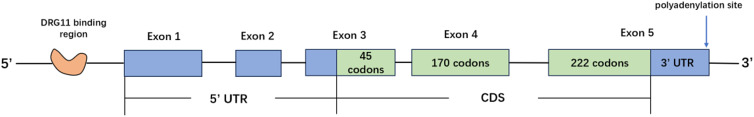
The main structure of human RGMb gene. RGMb gene is composed of DRG11 binding region and five exons. The coding regions are indicated by the green boxes, and the non-coding regions are represented by the blue boxes. The DRG11 binding region is located upstream of the gene, which is the promoter region of RGMb. The polyadenylation site is depicted by a vertical arrow.

RGMb, also known as Dragon, was originally identified by using a genomic screen of genes in the embryonic dorsal root ganglia that are regulated by the transcription factor DRG11 (Refs 7, 23). To date, RGMb genes have been identified in eight mammalian and seven non-mammalian vertebrates. It is a single-copy gene and contains a conserved chromosomal locus. The human RGMb gene is ~25 kb in length, located at 5q15 and contains 5 exons, including two 5′ non-coding exons (×Fig. 1). The 5′ non-coding exons (exon 1 and exon 2) include about 406 nucleotides of a ~524 nucleotide 5′ UTR of RGMb mRNA, and the remaining 118 nucleotides of the 5′ UTR are located in exon 3, which encodes the first 45 codons. The next 170 codons and the remaining 222 codons are located on exons 4 and 5, respectively. The 3′ UTR of 308 nucleotides found in exon 5 includes a single polyadenylation signal. The 363 bp DNA fragment at 750 bp upstream of the gene represents the DRG11 binding region and is located in the promoter region of the RGMb gene. So far, only three RGMb exons have been identified in the mouse genome, which correspond to 3–5 exons of the human RGMb gene, respectively. The 3′ UTR of the mouse RGMb mRNA is about 2.5 kb in length, encoded by exon 3, which is longer than the human counterpart (Ref. 24). In contrast, to date only coding information of RGMb is available for zebrafish. Unlike human, the coding region of RGMb in zebrafish is found within three distinct exons (Ref. 7).

The initial cloning of mouse RGMb cDNA resulted in a predicted protein of 438 amino acids, which is 89% identical to human RGMb and 65% identical to zebrafish RGMb, with 437 and 436 amino acids, respectively (Ref. 24). The molecular structural sequence of RGMb contains an N-terminal signal peptide of ~50 residues, and a C-terminal GPI anchor domain of ~35 amino acids. Other recognizable protein elements in RGMb include hydrophobic domain of unknown function and a vWF-type D domain that contains a putative internal proteolytic cleavage site. Researchers have confirmed that RGMb is localized on the lipid rafts of the cell membrane, and only one single-stranded RGMb is attached to the outer surface of the cell membrane (Refs 5, 25, 26). Unlike RGMa and RGMc, RGMb does not contain a conserved RGD (Arg-Gly-Asp) motif that is associated with cell adhesion. In fact, the adhesion of RGMb to cells mainly depends on the homophilic interaction (Refs 7, 27, 28). RGMb is also considered to be a glycoprotein, encoding two asparagine-linked glycosylation sites. The mature RGMb contains 14 cysteine residues and is a disulphide-binding protein, but the disulphide-binding mode needs to be further research.

## Localization and expression of RGMb

The repulsive effect of RGM on neuronal axons was first demonstrated in chicken nervous system development. Studies have shown that RGMb is a mammalian homolog of chick RGM (Refs 21, 29). The gene encoding RGMb was confirmed to be regulated by DRG11 and co-existed with DRG11 mRNA in dorsal root ganglia and spinal cord. DRG11 is a homeobox transcription factor expressed in embryonic DRG and dorsal horn neurons, which plays a role in a developing sensory circuit (Ref. 6). Samad *et al*. named RGMb gene ‘DRG11-response axonal guidance and outgrowth of neurite’ (DRAGON), indicating that it is turned ‘on’ in the ‘DRG’. Intriguingly, RGMb mRNA is expressed earlier than DRG11 during nervous system development. Meanwhile, RGMb is also expressed in other regions of the nervous system where DRG11 does not exist, suggesting that RGMb expression may be regulated by other mechanisms (Ref. 7). The results of in situ hybridization experiments found that RGMb mRNA is strongly expressed in several regions associated with the mouse embryonic central nervous system (Refs 21, 30, 31). In fact, RGMb is continuously expressed in some regions after birth, but the expression level is lower than that during development. Western blotting showed that RGMb was expressed in HEK293T cells, and its protein molecular weight was 50–55 kD. A similar slightly lower band was later found in extracts from neonatal and adult DRG and DRG primary cultured cells after incubation with phosphatidylinositol-specific phospholipase C, which cleaves the GPI anchor. These findings suggest that endogenous RGMb is proteolytically cleaved and only a single-chain RGMb species is attached to the outer surface of the cell membrane. Besides, immunohistochemistry revealed that RGMb protein is located in the region where RGMb mRNA expression has been found, including the DRG, spinal cord, brain and the retinal ganglion cells of developing mice (Ref. 7).

In 2005, after proving that RGMb is a co-receptor of BMPs, Xia *et al*., using BMPs as a clue, confirmed that BMPs and RGMb are highly expressed in the reproductive system. In addition to reproductive organs such as testis, epididymis, ovary, uterus and a variety of germ cells, RGMb also exists in pituitary cells that regulate reproductive organs and some tumour cells derived from the reproductive system, such as Ishikawa, HeLa, MCF-7, etc. In spermatogonia, the expression level of RGMb is positively correlated with gonadotropin (Refs 5, 14). Moreover, it was found that BMPs and RGMb are widely co-localized in the urinary system. RGMb was highly expressed and located in the apical membrane of epithelial cells in the rough segment of ascending branch, distal convoluted tubules and collecting duct of mouse medullary loop, but was lowly expressed in proximal tubules and glomeruli (Ref. 32). Since RGMb has been shown to be localized in macrophages, the authors believe that RGMb may be closely related to the immune system function (Refs 9, 33). In addition, RGMb was found to be involved in the development of spinal cord, sarcomere and skeletal muscle (Ref. 34). More recent data suggest that RGMb was also expressed in the heart, liver, lung, pancreas, embryo intestine, ganglion cells of the small intestine and colon and the crypts except pannet cells of rodents (Refs 30, 33, 35, 36).

## Molecular signalling pathways related to RGMb

Samad *et al*. found that the expression pattern of RGMb during development in mice and Xenopus was similar to that of BMP receptors (Ref. 26). Thus, they investigated the role of RGMb in BMP signalling and revealed that RGMb is a BMP co-receptor that enhances BMP signalling at the cell membrane. RGMb exists on lipid rafts and is a GPI-anchored protein without a transmembrane domain. RGMb can directly bind to BMP-2 and BMP-4, but the affinity with other ligands is relatively low. Although it can neither change the intracellular kinase activity of the BMP receptor, nor alter the binding affinity of the BMP receptor to the activated Smad protein, RGMb can promote the assembly and trafficking of the receptor complex in microdomains by localizing to lipid rafts, making the complex binding more stable. This allows BMP signalling to react with low levels of ligand and thus enhances signal transduction (Refs 5, 37, 38). It is noteworthy that RGMb plays multiple roles in embryos to enhance BMP signalling at specific times and locations. RGMb can enhance the sensitivity of cells to low BMP ligands to varying degrees, resulting in lower levels of BMP ligands. And this low level of ligand favours the BMP spatial gradient established in the embryo. Expression of RGMb enable cells to respond earlier or greater to specific levels of BMP than cells that do not express RGMb (Ref. 26). Moreover, the transfection of RGMb cDNA alone can promote the BMP signalling, however, this effect is blocked by the endogenous antagonist Noggin, which sequesters the endogenously expressed BMP ligands. Similar to Noggin, the Fc fragment of RGMb can also inhibit the BMP signalling pathways by binding to BMP ligands (mainly including BMP-2 and BMP-4) and competitively inhibiting ligand binding to cell surface type I and type II receptors. The above results suggest that the action of RGMb enhancing BMP signalling is BMP ligand-dependent. However, Kanomata *et al*. found that RGMb can inhibit BMP signalling even in the absence of ligand, which conflicts with previous studies that RGMb is ligand-dependent (Ref. 39). They speculate that there may be a new cell membrane molecule that binds to RGMb, which blocks the transcription of the Smad. Nevertheless, the suppression mechanism and related biological functions require further research.

**Figure 2.**
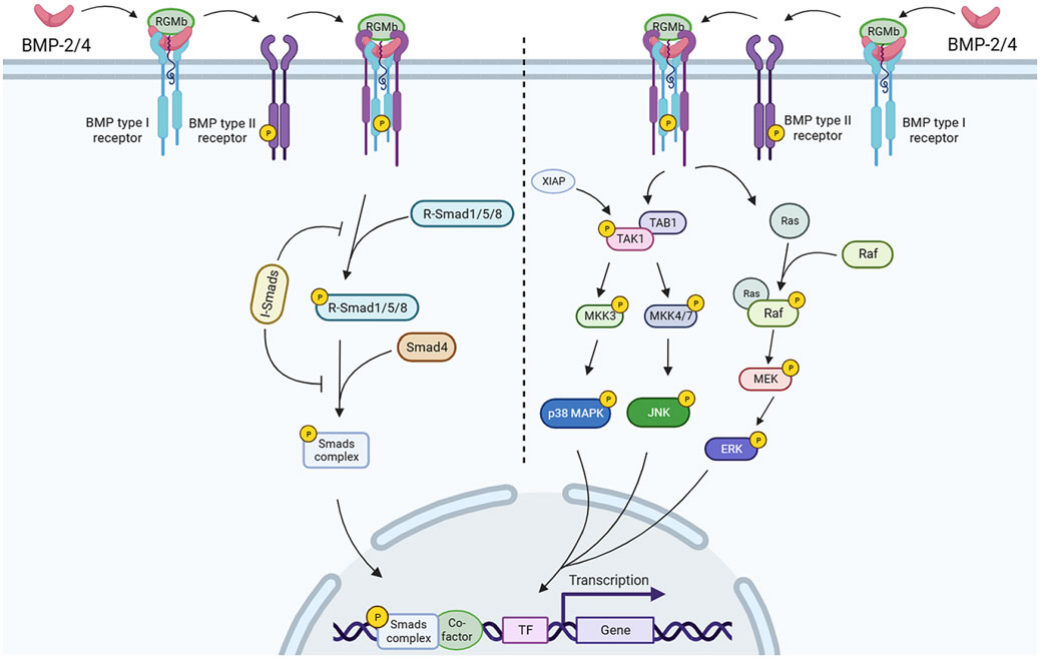
RGMb mediates BMP signal transduction pathway through two pathways. As a BMP co-receptor, RGMb can bind to BMP-2 or BMP-4, which preferentially bind to type I BMP receptors and then recruit type II BMP receptors. The type II BMP receptor is constitutively active and phosphorylates the type I BMP receptor. In Smad-dependent BMP signal transduction pathway (left), phosphorylated R-Smad1/5/8 form a complex with Smad4 and translocate into the nucleus. I-Smads negatively regulate BMP signal transduction by inhibiting receptor-mediated R-Smads phosphorylation and interfering with the formation of R-Smads and Co-Smads complexes. In Smad-independent BMP signal transduction pathway (right), RGMb directly binds to BMPs and mediates p38 MAPK, JNK and ERK phosphorylation by activating TAK1 and RAF, and that result in downstream target gene transcription.

BMPs are the largest subfamily of the transforming growth factor-*β* (TGF-*β*) superfamily, containing at least 20 members (Refs 40, 41, 42, 43). RGMb is known to enhance the BMP signalling pathway (×Fig. 2), but has no effect on TGF-*β* signalling. On the cell surface, BMP ligands bind to two different types of receptors, type I and type II transmembrane serine/threonine kinase receptors. Both receptors are composed of N-terminal extracellular ligand binding domain, transmembrane region and C-terminal serine/threonine kinase extracellular domain. Among the known TGF-*β* receptors, ACTR-IIA, ACTR-IIB and BMPR-II in type II receptors and ALK1, ALK2, ALK3 and ALK6 in type I receptors can bind to BMP ligands. In the canonical pathway, BMPs need to bind to BMP type I receptors before binding to type II receptors, which is different from TGF-*β* that preferentially binds to TGF-*β* type II receptors (Ref. 44). The reason why RGMb promotes BMP signal transduction is due to the increased availability of BMP type II receptors. RGMb normally enhances the utilization of ACTR-IIA by BMP-2/4. In the absence of RGMb, BMP-2/4 preferentially acts on BMPR-II. BMPs form heteromeric receptor complex with type I and type II serine/threonine kinase receptors and this complex is defined as BMP-induced signalling complexes (Ref. 45). BMP type II receptors have constitutive activity and phosphorylates the GS region of BMP type I receptors. Subsequently, the serine kinase of type I receptor phosphorylates the specific Smad proteins (Refs 46, 47). The smad proteins are intracellular signalling molecules composed of receptor-regulated Smads (R-Smads), common-mediator Smads (Co-Smads) and inhibitory Smads (I-Smads). R-Smads contain Smad1, 2, 3, 5 and 8, and its C-terminal Ser-Ser-Val/Met-Ser sequence (SSXS motif) can be activated by TGF-*β* type I receptors and form transient complex with receptors. Among these R-Smad proteins, only Smad1, 5 and 8 are activated by BMP type I receptors and share highly similar sequences. In mammals, only Smad4 has been confirmed to belong to Co-Smads. Smad4 can bind to activated R-Smads and form a heterodimer complex to transfer into the nucleus, thereby activating BMP signalling pathways (Refs 48, 49, 50). R-Smad proteins contain two different domains: MAD homology 1 (MH1) domain and MAD homology 2 (MH2) domain. The MH1 domain is a DNA binding region involved in the transcriptional activity of Smad complex. The MH2 domain contains a SSXS motif for c-terminal phosphorylation, which is the effector region of Smad protein and is responsible for the formation of Smad homodimers and their binding to BMP receptors. In the non-activated state without receptors, the regions of MH1 and MH2 interact each other to form self-inhibition. As the c-terminus of the MH2 domain is phosphorylated, the interaction between the MH1 and MH2 domains is terminated, and R-Smads are activated and released from the structure. R-Smads form a complex with Smad4 and can be translocated into the nucleus. I-Smads include Smad6 and Smad7, whose role is to block receptor-mediated R-Smad phosphorylation or interfere with the formation of R-Smad and Co-Smad complex, and negatively regulate BMP signal transduction (Refs 51, 52). Beside the canonical pathway, BMPs can also activate Smad-independent signal transduction systems. BMP activates the mitogen-activated protein kinase (MAPK) signalling pathway through the association of BMP receptor type I with TGF-*β* activated kinase (TAK1), TAK1 binding protein and Xenopus inhibitor of apoptosis (Ref. 53). Then TAK1 induces MKK3, a MAPK kinase (MKK), to activate the p38 MAPK pathway (Refs 54, 55). On the other hand, TAK1 can also activate c-Jun N-terminal kinase (JNK) through MKK4/7 (Ref. 56). Both p38 MAPK and JNK can be transferred to the nucleus to regulate the transcription factors ATF2 and c-Fos/c-Jun of BMP target genes. Besides, the activation of extracellular signal-regulated protein kinase (ERK) signalling pathway partially dependent on BMP has been confirmed. As a G protein, Ras binds to the amino terminus of Raf, a serine/threonine protein kinase, and activates Raf through an unknown mechanism. Raf can phosphorylate MAPK/ERK kinase (MEK) and then MEK transphosphorylates ERK. Eventually, ERK is activated and transferred to the nucleus (Ref. 57). The mechanism by which BMP receptors activate TAK1 and ERK remains to be further explored. In addition, TAK1 has been shown to mediate phosphorylation of Smad1 c-terminal serine residues in chondrocytes, suggesting that TAK1 can also act as an upstream kinase of R-Smad (Ref. 58).

In addition to the traditional BMP signalling pathway, RGMb also exerts biological functions through several other mechanisms. RGMb has a repulsive guiding effect on neurons, and this effect is achieved by the binding of neogenin molecules (Ref. 59). Neogenin is a member of the immunoglobulin receptor superfamily and expressed in the developing and adult nervous system. It is a homologue of deleted in colorectal cancer, a netrin receptor that is involved in axon guidance and cell survival (Refs 60, 61, 62). DRG axons in chicks do not express endogenous neogenin; however, they can be converted to reactivity by forced expression of neogenin (Ref. 63). In 2010, Zhou *et al*. found that neogenin promoted the BMP signalling pathway, and the triangle relationship of RGMb–BMPs–neogenin interaction emerged (Ref. 64). Nevertheless, Hagihara *et al*. showed that neogenin can activate RhoA, inhibit Smad1/5/8 phosphorylation and ultimately restrain BMP-induced osteoblast differentiation (Ref. 65). Based on these results, whether neogenin plays a role in promoting or inhibiting BMP signalling pathway is still inconclusive. Indeed, the extracellular region of RGMb contains a motif that can undergo autocatalytic cleavage. Studies have shown that the complete localization of RGMb on the cell membrane is a prerequisite for RGMb as a BMP co-receptor, whereas the intercellular RGMb is cleaved and acts on neogenin in the form of ligands (Refs 5, 17, 26, 27, 39, 66). As the PD-L2 receptor, however, RGMb has also been found to form supercomplexes with BMP, neogenin and PD-L2 when it is localized to the cell membrane through GPI-anchored proteins. In this supercomplex, PD-L2 is thought to indirectly affect BMP and neogenin signalling pathways by binding to RGMb. These inconsistent results indicate that the coordination between RGMb–neogenin and BMP–RGMb is still unclear.

## Cell biological functions

**Figure 3.**
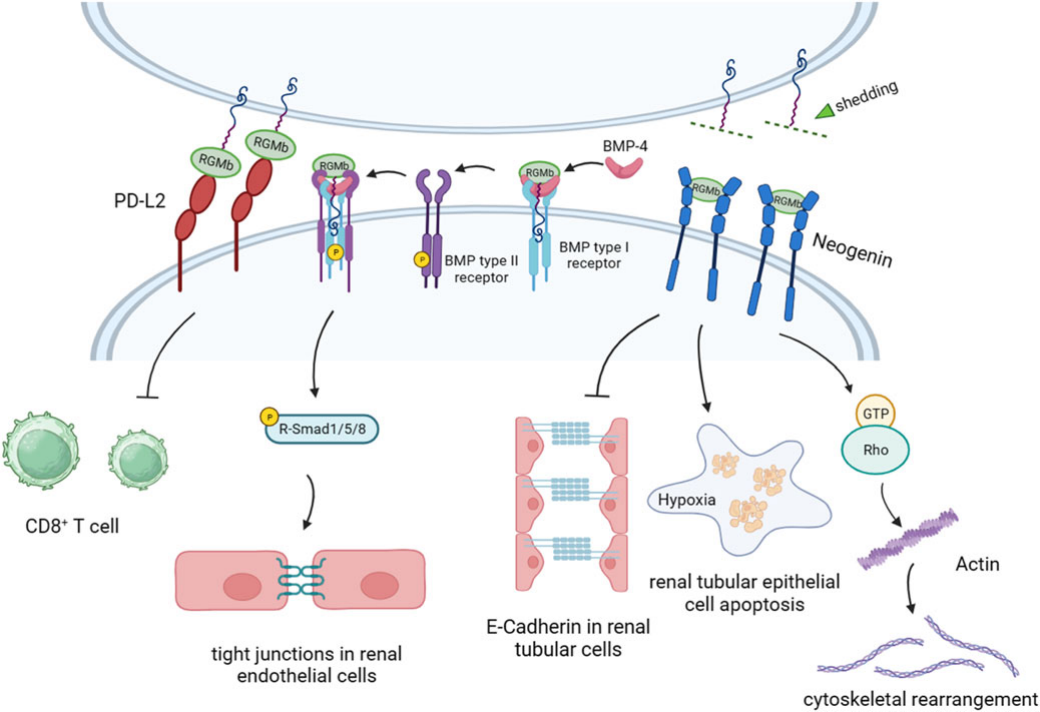
RGMb regulates downstream signalling pathways and cell functions by targeting different molecules. On one hand, RGMb inhibits epithelial E-cadherin expression and promotes hypoxia-induced cell apoptosis through neogenin in renal tubular epithelial cells. On the other hand, RGMb binds to the endogenous ligand BMP-4 in kidney epithelial cells, which significantly promotes intercellular tight junctions and enhances transepithelial resistance through the Smad1/5/8 pathway. Furthermore, PD-L2 overexpressed on dendritic cells interacts with RGMb on the surface of CD8^+^ T cells and inhibits CD8^+^ T-cell response. It's different that the intercellular RGMb directly binds to the cell surface receptor neogenin, and triggers the structural rearrangement of the actin cytoskeleton after cell rejection mediated by the small GTPase Rho family.

The widespread expression of RGMb in tissues suggests that RGMb may play important biological roles in a variety of tissues (Fig. 3). It has been reported that RGMa has the function of repulsive and axon-specific guidance activity (Ref. 1). Recombinant RGMa can induce the collapse of the temporal growth cone in retinal ganglion cells at low nanomolar concentrations and guide the temporal retinal axon in vivo, but it does not induce the collapse of the nasal growth cone. Different from RGMa, RGMb could not lead to the collapse of the growth cone of embryonic DRG neurons, and had no repulsive effect on DRG neurons of embryos and pups. However, RGMb was expressed in DRG neurons and it could promote intercellular adhesion of neurons through homophilic interaction to achieve the function of regulating axonal rejection. In fact, as a member of the RGM family of membrane-associated GPI-anchored proteins, overexpression of RGMb in cells can significantly enhance the adhesion of isolated DRG neurons to these cells. RGMb was found to be comparable to the combination of poly-D-lysine and laminin in enhancing neuronal adhesion by quantifying the amount of DRG neuron adhesion on the coated plates (Ref. 7). Moreover, the complementary expression of RGMb and RGMa in the spinal cord and brain suggests that there may be an interaction between them. Similar to RGMa, RGMb can also inhibit the axonal overgrowth of cerebellar granule neurons in postnatal rats through the RhoA/Rho-kinase signalling pathway in vitro, but its expression in the surrounding tissues of the spinal cord increases when the spinal cord is injured (Refs 67, 68). These results suggest that RGMb may be able to inhibit axonal regeneration in the central nervous system of adult mammalian. Additionally, Jorge *et al*. found that RGMb is also involved in the development of trigeminal, facial and optic nerves (Ref. 34).

BMP signalling pathway is involved in embryonic development and maintenance of tissue homeostasis (Refs 69, 70). The functional loss of BMP signalling pathway elements is closely related to embryonic death (Refs 71, 72, 73), developmental malformations (Refs 74, 75, 76) and physiological dysfunction (Refs 77, 78, 79). As a BMP co-receptor, RGMb is involved in important biological functions in cells and tissues, which plays a dual role in BMP signalling pathway through Smad-dependent and Smad-independent pathways. On the one hand, RGMb is expressed throughout the cells associated with the reproductive system, and the expression level is dynamically regulated at different stages and in different parts, overlapping with the expression site of the BMP signalling system, indicating that RGMb plays an important role in mammalian reproduction (Ref. 14). Overexpression of RGMb mRNA in Xenopus embryos enhances the expression of mesoderm and endoderm tissue markers, promoted neuronal phenotype and inhibited neural ridge differentiation. RGMb can also promote neurite outgrowth and peripheral nerve regeneration through the BMP signalling pathway in vivo. In the presence of RGMb deletion or BMP inhibitors, BMP signalling is attenuated, which delays early axonal regeneration after sciatic nerve crush injury (Ref. 8). In BMP-responsive cell lines, overexpressed full-length RGMb can increase the activity of promoter–reporter genes containing a BMP-responsive transcriptional regulatory element (Refs 14, 26). These results indicate that RGMb has the ability to enhance BMP signalling pathway in vivo and in vitro. Furthermore, RGMb has significance in enhancing BMP signal transduction in renal tubular epithelial cells and maintaining normal renal physiological function. RGMb is reported to be expressed in the thick ascending limbs, distal tubules and collecting ducts in the kidney of mice. RGMb increases transepithelial resistance dependent on BMP signalling pathway. Xia *et al*. found that BMP4 normally signals through BMPRII in renal tubular epithelial cells, but RGMb enhances BMP4 signalling through ActRIIA. More specifically, RGMb mainly binds to the endogenous ligand BMP-4 in epithelial cells, which significantly promotes intercellular tight junctions and enhances the resistance of kidney epithelial cells through the canonical Smad (Smad1/5/8) pathway (Ref. 32). On the other hand, in C2C12 myoblasts, RGMb inhibited the activities of alkaline phosphatase and inhibitor of DNA binding 1 promoter induced by BMP-4 or constitutively activated BMP type I receptors, and this inhibition is dependent on the secreted form of the vWF-type D. Studies on macrophages revealed that RGMb can inhibit the expression of IL-6, and the loss of RGMb can lead to an increase in the expression of IL-6 and other inflammatory factors in macrophages, which affects the balance of the immune system. Notably, RGMb in macrophages regulates BMP signalling through two Smad-independent pathways, p38 MAPK and Erk1/2, but not Smad1/5/8. These results indicate that RGMb is an important regulator of immune cells and may provide new strategies for studying the mechanisms of immune diseases and inflammatory responses. Previous studies have found that mice RGMb knockout do not have obvious sensorimotor function or neurodevelopmental defects. However, neonatal mice with RGMb knockout are stunted and have increased mortality rate (Refs 9, 19), which suggests that RGMb also plays some unknown and important physiological functions in vivo.

So far, three different molecular family members, including netrins, RGMs and BMPs, have been found to bind to neogenin (Refs 63, 65, 80). RGM–neogenin plays multiple roles in the developing central nervous system, including retinal projection guidance (Ref. 81), neuronal cell proliferation, differentiation and death (Ref. 82), axon induction and neural tube closure (Ref. 83). It has been reported that RGMb can induce actin cytoskeletal rearrangement through neogenin and its downstream effector, the Rho family of small GTPases (Refs 17, 84). In addition to Rho, RGMb down-regulates the expression of epithelial E-Cadherin and increases hypoxia-induced apoptosis in tubular epithelial cells through the neogenin receptor (Ref. 10). Interestingly, Joseph *et al*. found that the RGMb–neogenin interaction is involved in the regulation of the number of progenitor cells and cell differentiation in the developing olfactory epithelium, suggesting that RGMb–neogenin may control the proportion of glial cells and neurons produced in the olfactory epithelium (Ref. 85). Notably, neogenin is expressed in the immature pyramidal cell layer with migration ability in hippocampus, while cells expressing RGMb constitute the periphery of limbic system, suggesting that RGMb may have the effect of restricting the migration of neogenin-positive neurons. Conrad *et al*. found that neogenin can promote the formation of dense cell colonies in 3T3-L1 cells. However, after transfection with RGMb, the cells dissociated from colony state to dispersed state, which shows that the intercellular adhesion decreased. Considering that RGMb can promote the adhesion between neurons in the spinal cord and peripheral nervous system, the dissociation of 3T3-L1 cells transfected with RGMb may be caused by the mutual exclusion of RGMb and neogenin. Some researchers have previously defined neogenin as a ligand-dependent receptor, but this conclusion has been increasingly questioned. Studies have shown that PC12 cells expressing neogenin alone could survive normally in the absence of RGMa and RGMb. Xia *et al*. also found that silencing of RGMb and/or neogenin can reduce the apoptosis rate of renal tubular epithelial cells (Refs 10, 84). These contradictory results make it difficult to determine the mechanism and physiological function of RGMb.

**Figure 4.**
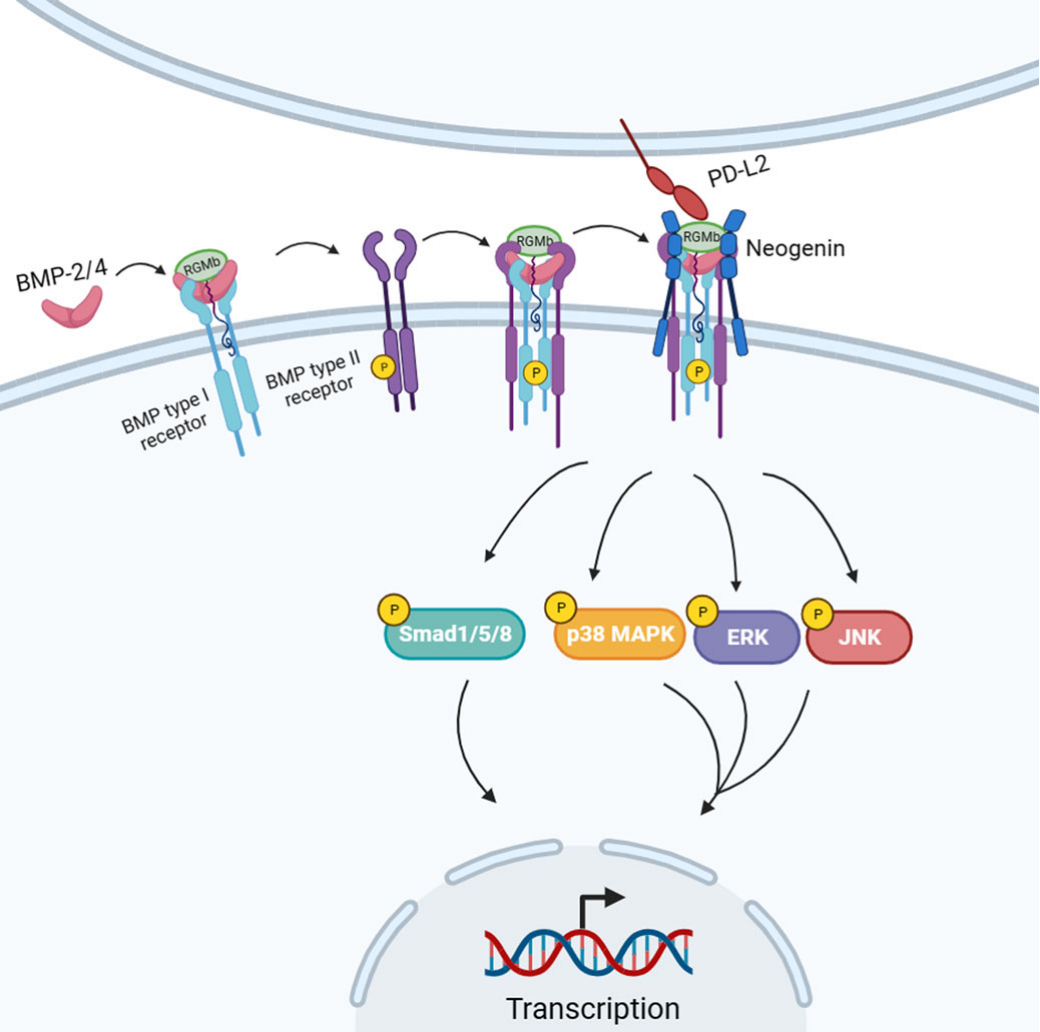
As a PD-L2 receptor, RGMb cooperates with receptors in the BMP and neogenin pathways to form a BMP–BMP receptor–RGMb–neogenin (BBRN) signalling supercomplex. In this supercomplex, the binding of PD-L2 to RGMb may affect the conduction of BMP and neogenin downstream signalling pathways.

Previous studies have shown that PD-L2 binds to PD-1 on T cells to inhibit T-cell activation, and the affinity of PD-L2/PD-1 is three times that of PD-L1/PD-1. PD-L2 and PD-L1 competitively bind to PD-1 due to their similar binding regions to PD-1. Although the affinity of PD-L2 is higher than that of PD-L1, its low expression level provides a competitive advantage for PD-L1/PD-1. Another binding protein of PD-L2 is RGMb, which has an affinity similar to PD-L2/PD-1. RGMb directly binds to PD-L2 to promote the formation of immune tolerance in the respiratory system. PD-L2 and BMP can bind to RGMb at different binding sites to form a BMP–BMP receptor–RGMb–neogenin (BBRN) signalling supercomplex and regulate downstream signalling pathways (Ref. 33) (Fig. 4). The discovery of different binding sites for PD-L2 and BMP-2/4 in RGMb reveals that RGMb is not only precisely regulated, but also may have an unknown complex signalling pathway. More recently, Joon *et al*. showed that PD-L2, which is highly expressed on the dendritic cells in lymph nodes of some intestinal bacteria, interacts with RGMb on CD8^+^ T cells and inhibits the immune response of CD8^+^ T cells (Fig. 3). In the host tumour microenvironment with adverse intestinal flora, RGMb may induce up-regulation of CD8^+^ tumour-infiltrating lymphocytes. They also found that antibodies targeting PD-L2/RGMb combined with anti-PD-1 or anti-PD-L1 therapy can overcome the resistance of PD-1 pathway inhibitor monotherapy in the context of dysbiosis (Ref. 86). Marine *et al*. believed that this study is very attractive compared to other reported mechanisms of immune checkpoints. This is because the authors found that down-regulation of the PD-L2–RGMb pathway is an immunomodulatory mechanism of the gut microbiota, which provides a new strategy for personalized intervention of immunodeficiency caused by dysmicrobialism (Ref. 87). In addition, RGMb can also interact with various molecules such as the immunoregulatory molecule CTLA-4 (Ref. 88) and PD-L2 mutant lysine with serine (K113S) at positive 113 (Ref. 18). These studies help us better explore the potential function of RGMb in immune activation.

## RGMb and diseases

Due to the broad biological functions of this protein, the role of RGMb in the development of human diseases, especially tumour-related diseases, is an area of increasing importance. The expression of RGMb in cancer may vary greatly depending on the expression of RNA or protein in different tissues (Table 1). Clinical data showed that RGMb expression was decreased in advanced (*P* = 0.017) and non-small-cell lung cancer (NSCLC) with vascular invasion (*P* < 0.001) and was associated with overall survival (39 *vs* 62 months, *P* < 0.001) was significantly associated with disease-related death (*P* = 0.015) (Ref. 89). In vitro and in vivo experiments revealed that the tumour suppressor effect of RGMb is achieved by inhibiting the Smad1/5/8 pathway. Some researchers found that circ_0001073 was lowly expressed in NSCLC tissues and cells. Circ_0001073 targets miR-582-3p via sponge adsorption to up-regulate the expression of RGMb, which inhibits the proliferation, migration and invasion of NSCLC cells and induces apoptosis (Ref. 13). In addition, the recent report has shown that RGMb is involved in the regulation of BMP2/4 signal transduction in NSCLC and regulates BMP2-mediated *ID* mRNA expression. It is worth mentioning that they believe that RGMb can partially regulate the epithelial-mesenchymal transition balance of NSCLC cells, however, RGMb is independent of PD-L2 in the execution of these regulatory functions (Ref. 90). As previously reported, the knockdown of RGMb also was studied in prostate cancer (Ref. 91). The results showed that the knockdown of RGMb significantly promoted the growth, adhesion and migration of prostate cancer cells. In gastric cancer tissues and cells, the expression of circ_0000419 and RGMb was down-regulated, and the expression of miR-300 was up-regulated. Circ_0000419 acts as a molecular sponge to adsorb miR-300, which targets RGMb and negatively regulates the expression of RGMb, further inhibiting the migration, invasion and glycolysis of gastric cancer cells (Ref. 92). However, RGMb expression was elevated in colon cancer tissues at both transcript and protein levels. Studies have shown that RGMb stimulates colon cancer progression through BMP4 and its downstream Smad1/5/8 and ERK1/2 pathways both in vitro and in vivo (Ref. 93). Besides, Li *et al*. firstly revealed that RGMb knockdown enhanced the proliferation, adhesion and migration of breast cancer cells (Ref. 94). RGMb knockdown could up-regulate Snai1, Twist, FAK and paxlin through enhanced Smad-dependent signalling, resulting in enhanced adhesion and migration. At the same time, Caspase-3 expression and activity were decreased by inhibiting the Smad-independent pathway JNK, which resulted in improved breast cancer cell survival. Recently, some researchers proposed that miR-93-5p promotes tumour migration and invasion by inhibiting the expression of RGMb in squamous cell carcinoma of the head and neck (Ref. 95). More recently, RNF4 is known to be required for BMP6 and its co-receptor RGMb mRNA expression (Ref. 96). The key role of RNF4–RGMb–BMP6 axis in osteogenic differentiation of human bone marrow mesenchymal stem cells and tumorigenesis has been reported. RNF4 deficiency can induce osteogenic differentiation of human bone marrow-derived mesenchymal stem cells. As downstream target molecules of RNF4, RGMB and BMP6, proteins play a key role in osteogenic differentiation of human bone marrow-derived mesenchymal stem cells. Statistical analysis showed that high levels of RNF4 and BMP6 were negatively correlated with the prognosis and disease-free survival of various sarcomas, while RGMb was positively correlated with RNF4 expression (Ref. 97).

**Table 1.** Expression of RGMb and LncRNA RGMb-AS1 in human tumour tissue compared with normal

Cancer type	Expression	Analysed level	Function	References
Non-small-cell lung cancer	Low	Protein/mRNA	Tumour suppressor	(Ref. 89) (Ref. 13)
	High	AS-LncRNA	Oncogenic	(Ref. 11)
Prostate cancer	High	Protein/mRNA	Tumour suppressor	(Ref. 91)
Gastric cancer	Low	Protein/mRNA	Tumour suppressor	(Ref. 92)
Colorectal cancer	High	Protein/mRNA	Oncogenic	(Ref. 93)
Breast cancer	–	–	Tumour suppressor	(Ref. 94)
Squamous cell carcinoma of the head and neck	–	–	Tumour suppressor	(Ref. 95)
Papillary thyroid carcinoma	High	AS-LncRNA	Oncogenic	(Ref. 12)
Cervical cancer	High	AS-LncRNA	Oncogenic	(Ref. 101)
Gastric cancer	High	AS-LncRNA	Oncogenic	(Ref. 102)
Pancreatic cancer	High	AS-LncRNA	Oncogenic	(Ref. 103)
Glioma	High	AS-LncRNA	Oncogenic	(Ref. 99)
Osteosarcoma	High	AS-LncRNA	Oncogenic	(Ref. 104)
Laryngeal squamous cell carcinoma	High	AS-LncRNA	Oncogenic	(Ref. 100)
Nasopharyngeal carcinoma	Low	AS-LncRNA	Tumour suppressor	(Ref. 106)
Hepatocellular carcinoma	Low	AS-LncRNA	Tumour suppressor	(Ref. 105)

In addition to elucidating the regulatory mechanism and impact of RGMb in cancer, the role of this protein coding gene in other pathologies has been studied. For example, Shi *et al*. found that RGMb plays an important role in maintaining the balance of intestinal flora. RGMb deficiency significantly altered gut microbiota diversity and contributed to dysbiosis. RGMb deficiency-mediated loss of Prevoteaceae in mice may be more susceptible to intestinal inflammation. RGMb is mainly expressed by bronchial epithelial cells, activated eosinophils and interstitial macrophages, whereas lung dendritic cells express PD-L2. Blockade of the RGMb-PD-L2 interaction significantly impairs the development of respiratory tolerance by interfering with the initial T-cell expansion required for respiratory tolerance. In addition, cells in the inflamed lung express the IL-25 receptor, an important cytokine in the airway inflammatory response. Blockade of RGMb with anti-RGMb mAb can reduce IL-25 production by modulating the RGMb–neogenin axis, and subsequently effectively block the development of airway inflammation and airway hyperreactivity. Deletion of RGMb exacerbates acute kidney injury. It has been shown that loss of RGMb in renal tubular cells increases the expression of mixed lineage kinase domain-like protein (MLKL) on the apical membrane of proximal tubules during renal injury. RGMb inhibits necroptosis in proximal tubular cells by preventing membrane-associated MLKL. The similar result revealed by Liu *et al*. showed that compared with wild-type mice, RGMb^+/−^ mice had reduced renal epithelial cell apoptosis and milder tubular damage. RGMb is up-regulated at the site of spinal cord injury in adult rats. Like RGMa, RGMb inhibits axonal regeneration by participating in the RhoA/Rho kinase signalling pathway, and RGMb is also a myelin-derived axonal growth inhibitor. There are also data showing that RGMb can stimulate the BMP signalling pathway and has a positive regulatory effect on neurite extension in vitro and early axon regeneration after nerve injury in vivo. Conversely, RGMb depletion delayed early axonal regeneration after sciatic nerve crush injury by inhibiting BMP. In addition, RGMb secreted by neurons is considered to be a key regulator of melanocytes and may be involved in pigment disorder diseases. It can not only induce melanocyte morphogenesis and melanin production, but also regulate melanocyte activity and promote dark vesicle transport (Ref. 98).

## LncRNA RGMb-AS1 in cancers

Long non-coding RNAs (lncRNAs) refer to non-coding RNAs longer than 200 nucleotides that do not have the ability to encode proteins. The RGMb Antisense RNA 1 (RGMb-AS1) gene is located on human chromosome 5q21.1, and its transcription product is lncRNA RGMb-AS1 with a length of 2053 bp. It is worth noting that the role of lncRNA RGMb-AS1 in a variety of diseases has also attracted a wide spread attention. The expression level of lncRNA RGMb-AS1 in different human tumour tissues has been described (Table 1). LncRNA RGMb-AS1 was initially found to be highly expressed in NSCLC tissues compared with adjacent normal tissues. The overexpression of lncRNA RGMb-AS1 was associated with the degree of differentiation, lymph node metastasis and TNM stage of lung cancer patients. Down-regulation of lncRNA RGMb-AS1 inhibited the proliferation, migration and invasion of lung cancer cells, and blocked cell cycle progression (Ref. 11). In 2018, Zhang *et al*. found that the transcription factor E2F1 can promote the transcription of lncRNA RGMb-AS1 by binding to the promoter region of lncRNA RGMb-AS1 in papillary thyroid carcinoma (PTC). The highly expressed lncRNA RGMb-AS1 promotes the proliferation, invasion and migration of PTC (Ref. 12). Furthermore, it was found that the lncRNA RGMb-AS1 was up-regulated in various malignant tumours, such as cervical cancer, gastric cancer, pancreatic cancer, glioma, osteosarcoma and laryngeal squamous cell carcinoma (Refs 99, 100, 101, 102, 103, 104). In these tumours, lncRNA RGMb-AS1 normally acts as a competing endogenous RNA to regulate gene expression through competitively binding to microRNA, which contributes to tumour progression.

In contrast, the expression of lncRNA RGMb-AS1 was down-regulated in nasopharyngeal carcinoma and hepatocellular carcinoma (Refs 105, 106). Overexpression of lncRNA RGMb-AS1 can inhibit the progression of tumours, so lncRNA RGMb-AS1 may be a good prognostic indicator for these tumours. The researchers demonstrated that the tumour suppressor effect of lncRNA RGMb-AS1 in nasopharyngeal carcinoma cells can be mediated through direct binding to FOXA1. However, the specific mechanism of lncRNA RGMb-AS1 inhibiting tumour remains unclear.

## Conclusions and perspectives

In summary, RGMb, along with two other members of the RGM family, RGMa and RGMc, was originally studied in the field of the nervous system. During the last 20 years, RGMb was found to be expressed in various tissues and organs of the cardiovascular system, digestive system, respiratory system, urinary system, reproductive system and immune system. It is generally believed that RGMb plays a regulatory role in embryonic development, immune function, cell adhesion, tumorigenesis and injury repair through neogenin–Rho signalling pathway and BMP signalling pathway. Other molecules such as PD-L2 can also participate in the functional regulation of RGMb. PD-L2–RGMb has been shown to mediate anti-tumour immunity and is expected to provide a potentially effective immune strategy. However, the potential mechanism of direct or indirect interaction between these molecules and RGMb remains to be further studied.

So far, new insights have been provided into RGMb biological function and its role in some diseases. On one hand, RGMb affects the proliferation, invasion and migration of tumour cells. On the other hand, RGMb plays an important role in intestinal inflammation, kidney injury, spinal cord injury and respiratory tolerance-related diseases. RGMb-AS1 is one of the newly studied lncRNAs in recent years. LncRNA RGMb-AS1 is abnormally expressed in a variety of tumour diseases and usually used to regulate target genes by sponge adsorption of microRNA, which affects the progression of various tumours. Finally, the research on RGMb is not comprehensive, and there may be some unknown mechanisms of action, which are related to the occurrence and development of certain diseases. Therefore, these need to be further confirmed by more research work.

## Data Availability

Not applicable (the data in this review all came from the referenced articles).
